# Nutritional Ketosis Affects Metabolism and Behavior in Sprague-Dawley Rats in Both Control and Chronic Stress Environments

**DOI:** 10.3389/fnmol.2017.00129

**Published:** 2017-05-15

**Authors:** Milene L. Brownlow, Seung H. Jung, Raquel J. Moore, Naomi Bechmann, Ryan Jankord

**Affiliations:** ^1^Applied Neuroscience Branch, Warfighter Interface Division, Air Force Research Laboratory, Wright-Patterson Air Force BaseDayton, OH, USA; ^2^Research Associateship Program, National Research Council, National Academies of ScienceWashington DC, USA; ^3^Infoscitex, Inc.Dayton, OH, USA

**Keywords:** nutritional ketosis, behavioral performance, metabolism, hippocampus, ketone supplements, stress

## Abstract

Nutritional ketosis may enhance cerebral energy metabolism and has received increased interest as a way to improve or preserve performance and resilience. Most studies to date have focused on metabolic or neurological disorders while anecdotal evidence suggests that ketosis may enhance performance in the absence of underlying dysfunction. Moreover, decreased availability of glucose in the brain following stressful events is associated with impaired cognition, suggesting the need for more efficient energy sources. We tested the hypotheses that ketosis induced by endogenous or exogenous ketones could: (a) augment cognitive outcomes in healthy subjects; and (b) prevent stress-induced detriments in cognitive parameters. Adult, male, Sprague Dawley rats were used to investigate metabolic and behavioral outcomes in 3 dietary conditions: ketogenic (KD), ketone supplemented (KS), or NIH-31 control diet in both control or chronic stress conditions. Acute administration of exogenous ketones resulted in reduction in blood glucose and sustained ketosis. Chronic experiments showed that in control conditions, only KD resulted in pronounced metabolic alterations and improved performance in the novel object recognition test. The hypothalamic-pituitary-adrenal (HPA) axis response revealed that KD-fed rats maintained peripheral ketosis despite increases in glucose whereas no diet effects were observed in ACTH or CORT levels. Both KD and KS-fed rats decreased escape latencies on the third day of water maze, whereas only KD prevented stress-induced deficits on the last testing day and improved probe test performance. Stress-induced decrease in hippocampal levels of β-hydroxybutyrate was attenuated in KD group while both KD and KS prevented stress effects on BDNF levels. Mitochondrial enzymes associated with ketogenesis were increased in both KD and KS hippocampal samples and both endothelial and neuronal glucose transporters were affected by stress but only in the control diet group. Our results highlight the complex relationship between peripheral metabolism, behavioral performance and biochemical changes in the hippocampus. Endogenous ketosis improved behavioral and metabolic parameters associated with energy metabolism and cognition while ketone supplementation replicated the biochemical effects within the hippocampus but only showed modest effects on behavioral improvements.

## Introduction

In creased abundance of food choices has resulted in widespread consumption of convenient meals that often lack nutritional value, contributing to the epidemics of obesity, diabetes and metabolic disorders. Clinical studies indicate a strong association between stressful events with adiposity, increased body mass index and weight gain (Dallman et al., 2005; Block et al., 2009). These metabolic changes are due, in part, to higher circulating levels of glucose, insulin and insulin resistance induced by cumulative stress (Sinha and Jastreboff, 2013). In non-diabetic individuals, higher levels of peripheral glucose were predictive of cognitive decline within 5 years whereas in diabetic patients, higher glucose levels were related to increased risk of dementia (Crane et al., 2013).

In the brain, prolonged periods of physiological and psychological stress are associated with decreased glucose availability, leading to impaired decision making abilities, reaction time, changes in attention and memory and learning deficits (Baran et al., 2009; Yuen et al., 2012; Olver et al., 2015). In rodents, performance in challenging tasks reduced glucose availability in several brain areas, particularly in the hippocampus (McNay et al., 2000), with more demanding tasks resulting in greater decreases compared to less demanding tasks (McNay et al., 2001). Therefore, nutrient availability and the inability to effectively utilize alternative fuel sources may contribute to deteriorating performance during physically and cognitively taxing settings. This underscores the importance of optimizing metabolic interventions targeting the homeostatic control of brain networks involved in efficient energy utilization.

The beneficial effects of low carbohydrate, ketogenic diets (KDs) on metabolism have been extensively described (Vernon et al., 2004; Westman et al., 2007). KDs enhanced athletic outcomes (Phinney et al., 1983; Volek et al., 2016) and reversed motor deficits in a model of amyotrophic lateral sclerosis (Zhao et al., 2012). Recently, KDs have been considered a potential therapeutic approach to neurological disorders (Kashiwaya et al., 2000; Prins, 2008; Puchowicz et al., 2008; Kelley and Hartman, 2011; Kim et al., 2012; Choi et al., 2016). Its neuroprotective effects can be associated with the ability of ketone bodies to act as additional energy substrates (Keon et al., 1995), replacing glucose as the brain's main energy source (Cahill, 2006; Masino et al., 2009; Courchesne-Loyer et al., 2013). Furthermore, recent studies are unraveling ketone bodies' role as potent signaling molecules in the brain (Shimazu et al., 2013; Newman and Verdin, 2014a,b), leading to adaptive cellular responses to environmental stimuli such as fasting, exercise and dietary interventions (Marosi and Mattson, 2014).

Chronic use of KD can be challenging due to limitations such as restricted food choices, unbalanced macronutrient profile and gastrointestinal side effects. Therefore, the low compliance to this diet led to the development of supplements such as synthetic ketone esters that mimic ketosis achieved with KD or prolonged starvation (Veech, 2004; D'agostino et al., 2013; Kashiwaya et al., 2013). Nutritional ketosis resulting from adherence to KD is often referred to as endogenous ketosis in contrast to peripheral ketosis induced by dietary supplements, referred to as exogenous ketosis. Ketone supplementation caused rapid and sustained elevation of blood ketones (1–5 mmol/L β-hydroxybutyrate) for hours after oral administration (D'agostino et al., 2013; Kesl et al., 2016). If demonstrated that exogenous ketones are capable of mimicking the beneficial effects of KD, dietary supplementation may be used to augment cognitive and physiological performance, bypassing the need for such a strict nutritional regimen.

While KD-induced benefits have been described in disease models, cognitive augmentation in healthy individuals still warrant investigation. Moreover, we sought to investigate the role of endogenous or exogenous ketosis following challenges known to result in functional, morphological and cognitive impairments. For instance, persistent stress and elevated corticosteroids are known to suppress neurogenesis and expression of brain-derived neurotrophic factor (BDNF) in the hippocampus (Rothman and Mattson, 2013) in addition to atrophy of dendritic branching (Sapolsky, 1985) and neuronal loss (Watanabe et al., 1992).

Taken together, the role of ketones in augmenting performance and cognition in the absence of disorders has only recently been the subject of investigation and merely anecdotal evidence is currently available. Thus, our aim was to test the hypothesis that ketone bodies are capable of preserving cognition during performance of cognitively demanding tasks or following exposure to stressful events. We investigated whether endogenous or exogenous ketones modulated metabolic, behavioral and biochemical events relevant to cognitive performance in young rats with no underlying neurological or metabolic dysfunction.

## Material and methods

### Ethical approval

All animal handling and procedures were approved by the Wright-Patterson Air Force Base Institutional Animal Care and Use Committee in accordance with the NIH Guide for the Care and Use of Laboratory Animals.

### Animals

Adult, male Sprague-Dawley rats (6–8 weeks old) were purchased from Charles River (Wilmington, MA). Animals were allowed to acclimate to Wright Patterson Air Force Base (WPAFB) animal facility (7–10 days) prior to experiments. Throughout experimental procedure, subjects were singly housed in clear Plexiglas cages (10.5W × 10L × 8H in) with *ad libitum* access to food and water. Ambient housing conditions were controlled for temperature (18–24°C), humidity (30–70%), and a standard 12 h light/dark cycle (0600–1800). All experiments were performed during the light phase (between 0700 and 1600). Animals were handled routinely from the time of their arrival to minimize any effects of handling stress on experimental measurements.

### Experimental procedure

**Figure 1:**
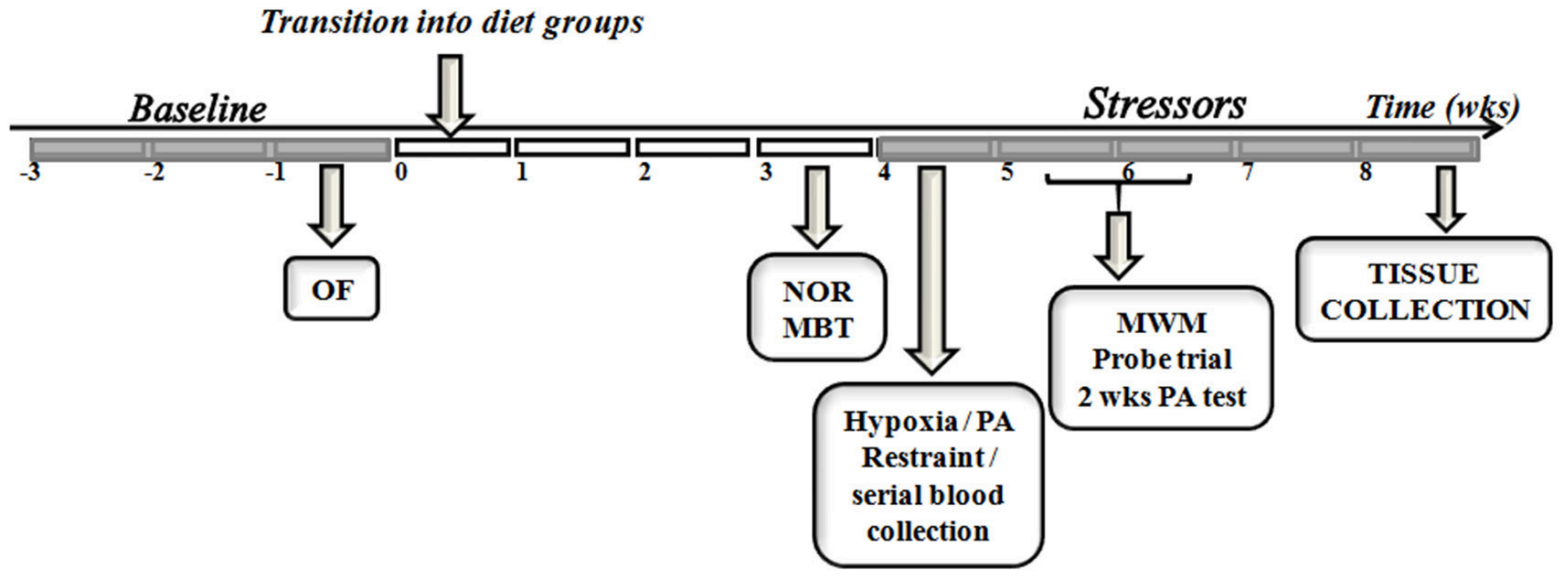
**Study timeline**. The experimental design used in the comparison between endogenous and exogenous ketones prior to and during exposure to control or chronic stress paradigm is depicted. OF, open field; NOR, novel object recognition; MBT, marble burying test; PA, passive avoidance; MWM, morris water maze; wks, weeks.

Figure 1 summarizes the experimental design adopted. All animals were individually housed before the commencement of the study. Food intake and body weight were assessed three times a week for 3 weeks before assignment into diet groups. Metabolic and behavioral data gathered during baseline (Table 1) were used to evenly distribute animals into dietary treatments (*n* = 20/diet). Peripheral blood was collected by tail clipping (between 0700 and 0900) and non-fasting levels of glucose and β-hydroxybutyrate (BHB) were measured using a glucose/ketone meter (Precision Xtra™, Abbott Laboratories, Abbott Park, IL).

**Table 1:** **Baseline parameters prior to assignment into dietary treatment groups**.

**Groups**	**Diets (*****n*****)**			
	**NIH-31 (20)**	**KD (20)**	**KS (20)**	***p*-value**
Body weight (g)	312.7 ± 4.3	321.1 ± 3.8	316.0 ± 4.4	0.36
Food intake (g/day)	27.1 ± 0.6	28.5 ± 0.5	27.7 ± 0.7	0.28
Estimated metabolic rate (FI/BW)	0.09 ± 0.004	0.09 ± 0.001	0.09 ± 0.002	0.57
Glucose (mg/dl)	119.6 ± 2.6	121.6 ± 3.78	119.0 ± 3.2	0.88
β-hydroxybutyrate (mM)	0.5 ± 0.03	0.5 ± 0.02	0.5 ± 0.05	0.99
**Open Field**				
Distance traveled (m)	21.0 ± 0.6	22.4 ± 0.6	22.4 ± 0.7	0.22
Speed (cm/s)	7.5 ± 0.2	8.0 ± 0.2	8.0 ± 0.2	0.27
Time Center/Total Time	0.03 ± 0.005	0.03 ± 0.004	0.03 ± 0.004	0.72

Subjects were progressively transitioned into new dietary groups, while access to previous diet was gradually reduced, allowing habituation to the new food. Animals were allowed to adjust to new diets for a 3 week period prior to behavioral testing. Subsequently, each diet group was subdivided into control or stress (*n* = 10/group) to investigate whether prolonged exposure to endogenous vs. exogenous ketones could mitigate challenge-induced detriments on behavioral performance. The stress paradigm adopted was chosen according the published studies on chronic variable stress on rodents (Herman et al., 2008; Jankord and Herman, 2008; Shea et al., 2015) and consisted of once-daily exposure to randomly assigned stressors. Restraint stress was performed by inserting the animals into a custom made well-ventilated, flat bottom clear plastic rodent restrainer for 1 h. Cold exposure consisted of placing cages (with no food, bedding or water) in a cold room at 4°C for 30 min. Constant motion was performed by placing cages onto an orbital shaker set at 100 rpm for 1 h. Novel housing consisted of placing animals into a novel cage (different dimensions – 16W × 20L × 8H in; different bedding with *ad libitum* access to food and water) overnight. Exposure to hypoxic conditions was performed by placing groups of rats in chambers with a solid lid containing inlet and outlets ports; ambient air was slowly replaced by high nitrogen, low oxygen gas mixture until it reached concentrations in the range of 8–12% O_2_. Animals were monitored throughout the hypoxia procedure (1 h). Stressors were randomly presented daily throughout the experimental paradigm. Serial blood collection was carried out during a 1 h restraint challenge to assess plasma levels of the stress hormones adrenocorticotrophic hormone (ACTH) and corticosterone (CORT). Moreover, all rats were fasted for 4 h, followed by *ad libitum* access to food for 2 h, after which food intake was calculated along with tail blood collection for measurements of postprandial levels of glucose, ketones and insulin. Animals were monitored daily for signs of distress and body weight was assessed three times a week to confirm normal growth rates for all animals.

### Diets

**Figure 2:**
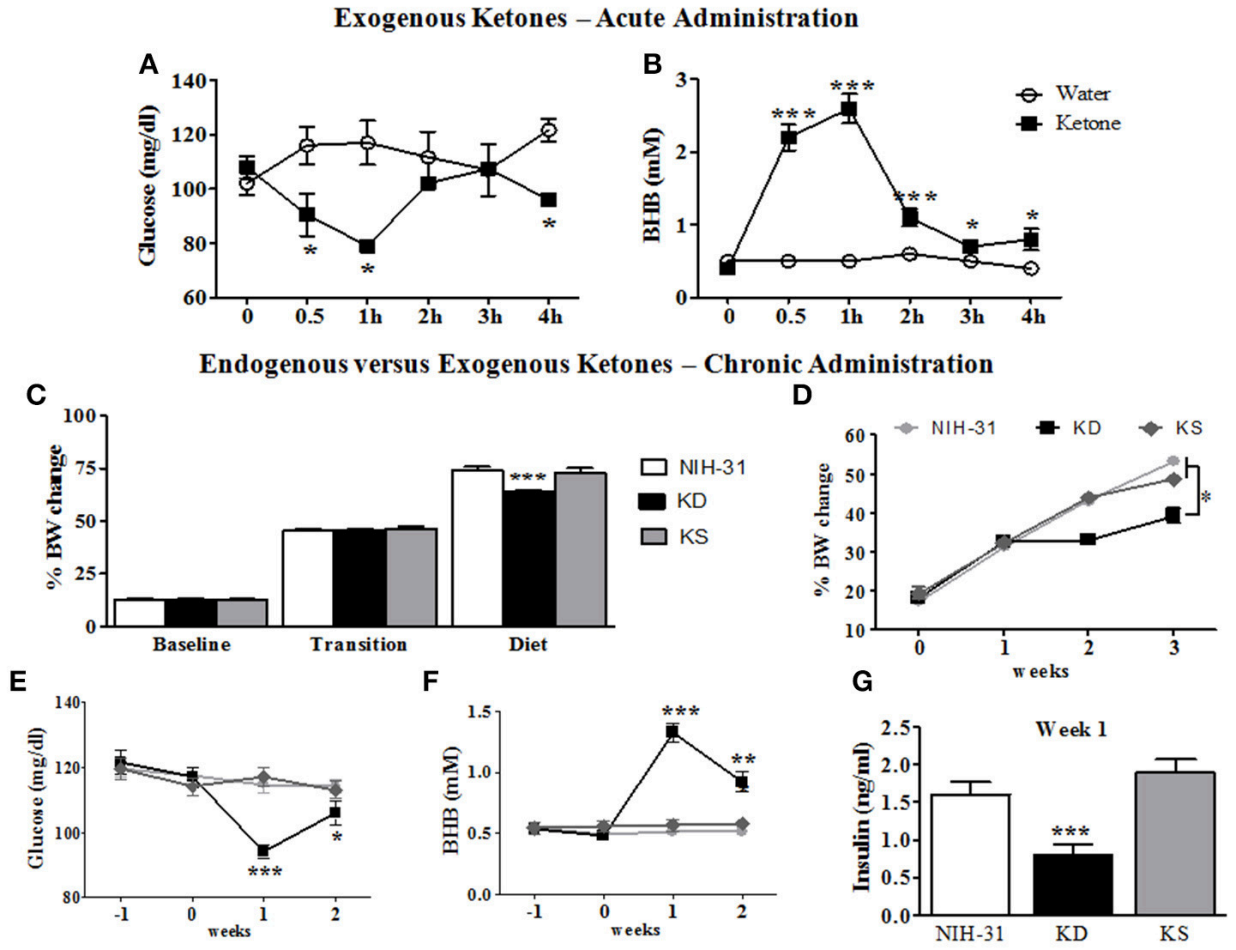
**Metabolic and behavioral changes induced by endogenous vs. exogenous ketosis**. Intragastric administration of exogenous ketones (mixture of KetoCaNa + Caprylic Triglyceride, CT) induces a reduction in blood glucose **(A)** while increasing and sustaining higher blood ketone levels for up to 4 h **(B)**. **(C)** No differences in body weight gain were observed during baseline assessment or transition into new diets whereas decreased body weight gain was observed in KD-fed rats following the start of dietary interventions. **(D)** KD-fed rats showed slower weight gain compared to control or KS diets after onset of chronic administration. **(E)** Blood glucose levels were significantly reduced in KD group 1 week after the start of dietary treatment. **(F)** KD feeding resulted in significantly elevated blood levels of the ketone body β-hydroxybutyrate (BHB) starting 1 week following dietary treatments. **(G)** Blood insulin levels at week 1 were significantly decreased in KD group. Data presented as mean ± S.E.M. (*n* = 6–8/group). ^*^p ≤ 0.05; ^**^p < 0.01; ^***^p < 0.001.

Three different diets were utilized for this study and the macronutrient distribution in each diet is detailed in Table 2. Briefly, the ketogenic diet (KD, Teklad, Madison, WI) consisted of a low carbohydrate, medium chain triglyceride diet (carbohydrates: 0.5%; proteins: 22.4%; fats: 77.1%; 4.7 kcal/g). This diet has been shown to successfully achieve ketosis in rodents (Brownlow et al., 2013), without introducing high amounts of omega-6 or hydrogenated fats when compared to the standard rodent NIH-31 diet (carbohydrates: 62.2%; proteins: 23.8%; fats: 14%; 3.0 kcal/g). We performed a preliminary study to ensure that a mixture of the commercially available ketone supplements Caprylic Triglyceride (CT, a medium chain triglyceride—MCT- purchased from Parrillo Performance, Fairfield, OH) and KetoCaNa (Prototype Nutrition, Urbana, IL; 20 g of KetoCaNa mixed into 100 ml of CT) successfully modulated peripheral levels of glucose and ketone bodies following intragastric administration at a 10 g/kg dose (Figures 2A,B). A mixture of ketone salts and MCTs was considered due to the finding of immediate induction of ketosis elicited by the salts coupled with a slower, more sustained increase following MCT processing in the liver, resulting in increased ketone production (Kesl et al., 2016). The proportion of supplements used was chosen based on suggestions by colleagues and experimental testing in pilot studies. We adjusted the final numbers based on how well the salts stayed in solution after being mixed with CT. Hence, the ketone supplemented diet (KS; carbohydrates: 59.5%; proteins: 21.8%; fats: 9.1%; ketone supplements: 10.2%; 4.3 kcal/g) consisted of adding the mixture of ketone supplements (described above) at a 10% concentration (w/v) into the powdered NIH-31 diet (CTL, Teklad, Madison, WI). Due to the soft consistency of both KD and KS, these diets could not be pelleted and were placed in jars on the bottom of the animal's cage. Food was replaced three times a week to ensure freshness and *ad libitum* consumption.

**Table 2:** **Nutritional information of diets used**.

**Diets**	**NIH-31**	**Ketogenic diet (KD)**	**Ketone supplementation (KS)**
**Ingredient**	**grams/kg**	**grams/kg**	**grams/kg**
Casein	210	300	210
L-Cystine	3	2.86	3
Sucrose	200	0	200
Maltodextrin	100	0	100
Corn starch	369	0	369
Cellulose (fiber)	40	245.31	40
MCT oil (medium chain triglycerides)	0	270	0
Flaxseed Oil	21	70	21
Canola Oil	19	60	19
Mineral mix Ca-P deficient (79055)	13.4	18.5	13.4
Calcium phosphate dibasic CaHPO4	7	8.5	7
Calcium carbonate CaCO3	7.3	10.75	7.3
40060 VM, Teklad	10	14	10
Ethoxyquin (Liquid)	0.1	0.08	0.1
β-hydroxybutyrate (BHB)	0	0	12.3
Caprylic triglyceride (CT)	0	0	80
Total	1,000	1,000	1,000
Protein, % of kcal	23.8	22.4	21.8
Carbohydrate, % of kcal	62.2	0.5	59.5
Fat, % by kcal	14	77.1	9.1
Ketone supplements	0	0	10.2
Vitamin mix, % of kcal	1.3	1.2	1.2
kcal/g	3.0	4.7	4.3

### Behavioral testing

The *open field* (OF) test was used as a standard test of general activity. Animals were placed for 5 min in an arena (40.5 × 45 × 36 cm Plexiglas) while their locomotor activity was monitored and quantified using EthoVision XT system (version 7.0.418, Noldus Information Technology, Leesburg, VT).

Short-term memory was assessed by the *novel object recognition* (NOR) test. Two identical objects were placed along the center line of the arena and animals were allowed to explore for 5 min. After each trial, the arena and objects were cleaned to minimize olfactory cues. Twenty four hours following the acclimation trial, one of the objects was replaced with a novel object. Animals were given a 5 min exploratory trial and working memory was evaluated by the percentage of exploration index (time exploring the novel object divided by the combined time spent exploring both novel and familiar objects multiplied by 100).

To assess memory acquisition and consolidation following hypoxia, *passive avoidance* (PA) was performed using classical conditioning chambers (Med Associates Inc., St. Albans, VT), as previously described (Sandusky et al., 2013). Briefly, animals in the stress groups were exposed for 1 h to hypoxic conditions (8–12% O_2_), prior to being placed in the brightly-lit side of the testing chamber for 30 s, after which a door opened, allowing entry to a dark chamber. Upon entry to the dark chamber on day 1, animals immediately received a mild foot shock (1.0 mA, 2s), ending their training trial and being subsequently placed back in the hypoxia chamber for 1 h prior to returning to their home cages. On day 2, animals were again placed in the brightly-lit chamber for 10s before the door was opened and latency to cross into the dark compartment (maximum of 10 min) after the door opened was taken as a measure of memory for the aversive experience.

The *Morris Water Maze* (MWM) tests spatial navigation and memory measured by the latency to find the escape platform (Morris, 1984). Water temperature was maintained at 19–23°C and extra-maze cues were placed on the walls. A clear platform (6 cm diameter) was located approximately 1.0 cm below the water in the southwest (SW) quadrant. Four alternating training days were completed, each with four 60 s trials with randomized starting positions. Inter-trial intervals were increased by testing all animals before the start of the next trial. On the first training day, animals that did not reach the platform within 60 s were gently guided to the platform. Probe trial was performed 48 h following the last (4th) training day, in which the platform was removed. Swim path, position, speed and latency to reach platform were recorded using EthoVision XT system (version 7.0.418, Noldus Information Technology, Leesburg, VT).

### Tissue processing

Rats were killed with pentobarbital (100 mg/kg) and transcardially perfused with 0.1M phosphate buffered saline (PBS). Interscapular brown adipose tissue (BAT), white adipose tissue (epididymal and perirenal fat pads), adrenals, thymus, and spleen were carefully harvested and weighed. Briefly, BAT was visually located after separating the skin on the back of the neck and dissected, cleaned from muscle and connective tissue prior to being weighed. Epididymal fat pads were dissected after opening the abdominal cavity, locating white adipose tissue in the inguinal anatomical region (slightly lateral to midline). After gently pulling the fat pad upward, the epididymus was visualized and gently separated from white adipose tissue so epididymal fat pad could be weighed. This was done on both sides. After locating the kidneys, perirenal fat pads were identified as the white adipose tissue located around the kidneys and extracted by carefully pulling and separating the fat from the kidney. Brains were collected immediately following perfusion; one hemisphere was dissected and immediately frozen on dry ice for biochemical analysis. The other hemisphere was immersion fixed in 4% phosphate-buffered paraformaldehyde for 24 h and cryoprotected in 30% sucrose solution for at least 24 h prior to sectioning on the coronal plane (25 μm thickness) on a sliding microtome with a freezing plate (Leica SM2010R). Sections were placed into cryoprotectant solution and stored at −20°C until further processing. Freshly frozen hippocampi were quickly minced and homogenized in RIPA homogenization buffer (pH 7.2) containing phosphatase and protease inhibitors (Sigma Aldrich, St Louis, MO) and soluble (cytosolic) fraction was separated following centrifugation at 20,000 rpm for 30 min. Pellets were then treated with Mem-Per Solubilization buffer, according to manufacturer's instructions (ThermoFisher Scientific, Waltham, MA) for extraction of membrane fraction. Hippocampal samples were used for further biochemical analysis by enzymatic or western blotting assays.

#### Biochemical analysis

All enzymatic assays were run in duplicate, analyzed within the same assay and performed according to manufacturer's instructions. Blood samples were centrifuged at 4,000 × g for 15 min at 4°C and plasma was stored at −20°C until processed. Plasma levels of ACTH and CORT were measured using a rat stress hormone magnetic multiplex bead assay (RSHMAG-69K, EMD Millipore, Temecula, CA). Insulin concentrations were determined by ELISA (Crystal Chem., Downers Grove, IL). Protein content in hippocampal homogenates was determined using BCA protein assay kit (Pierce, Rockford, IL); BDNF levels were quantified in the cytosolic fraction using ELISA (Bolster Biological, Pleasanton, CA) and normalized to total protein content. BHB concentration was measured using a commercially available kit (Cayman Chemicals, MI).

#### Western blotting

Following tissue homogenization and determination of protein concentration, equal amounts of proteins (12 μg/well) were loaded in each well of a 4–20% SDS PAGE gel and transferred onto a 0.2 μm pore size nitrocellulose membrane. Membranes were blocked in 5% blocking solution (Bio-Rad Laboratories, Inc. Hercules, CA) in 0.01% PBS-Tween and immunoblotted overnight (4°C on shaker) with different primary antibodies. Blots were washed with 0.01% PBS-T and incubated with horseradish peroxidase-conjugated goat anti-rabbit IgG secondary antibody (ThermoFisher, Waltham, MA) for 1 h at room temperature. After incubation, membranes were washed before visualization using enhanced chemiluminescence (SuperSignal West Femto, ThermoFisher Scientific, Waltham, MA). Primary antibodies used were all rabbit polyclonal: BDH1 (Proteintech, 1:1,000); ACAT1 (Proteintech, 1:1,000); GLUT1 (Proteintech, 1:1,000); GLUT3 (Abcam, 1:2,000); COX IV (Abcam, 1:1,000) and β-actin (Invitrogen, 1:5,000). Band intensities were quantified by densitometry analysis using ImageJ software; corrected for background intensities and normalized to levels of β-actin (soluble fraction) or cytochrome oxygenase IV (COX IV, for mitochondrial enzymes) used as loading controls.

### Statistical analysis

Statistical analysis was carried out using SigmaPlot (SigmaPlot 11.0, San Jose, CA) by ANOVA followed by Fisher's Least Significant Difference (FLSD) *post-hoc* analysis for all comparisons performed. One-way ANOVA was performed with diet as independent variable; two-way ANOVA with diet and stress or time points as independent variables and three-way ANOVA with diet, stress and test session (training, testing, or 2 week test for passive avoidance test and testing days for MWM). One-, two-, or three-way repeated measures (RM) ANOVA were used when necessary and are indicated in corresponding results section. Statistical significance was established with *p* ≤ 0.05 for all tests. Graphs were generated using Graph Pad Prism 5.01 (La Jolla, CA).

## Results

### Acute administration of exogenous ketones rapidly changed blood glucose and ketones

An initial time course experiment was performed to ensure that ketone supplements successfully induced and maintained ketosis in rodents. Administration of exogenous ketones by oral gavage reduced blood glucose within the first 30 min (95.3 mg/dl; two-way RM ANOVA treatment and time interaction, *p* = 0.02, Figure 2A), when compared to animals gavaged with water (116.0 mg/dl). Peripheral levels of BHB seemed to peak 1 h following administration and were significantly elevated for the duration of testing (ketone: 2.6 mM vs. water: 0.5 mM; two-way RM ANOVA main effects of treatment: *p* = 0.0002; time: *p* < 0.0001 and treatment and time interaction: *p* < 0.0001; Figure 2B).

### KD, but not KS, impacted body weight and blood levels of glucose, ketones, and insulin

Body weight data collected was averaged for different experimental phases (3 weeks of baseline, transition week and 3 weeks of dietary interventions) and percentage of body weight change from initial week (wk -3) was calculated (Figure 2C). No differences in body weight gain were observed during the initial 3 weeks of baseline assessments (one-way RM ANOVA, *p* = 0.64) or during the transition week (*p* = 0.80) and a main effect of diet (one-way RM ANOVA, *p* < 0.001) was observed following the start of diet treatments. Body weight gain was significantly slower in KD-fed rats when compared to both control and KS diets (FLSD, *p* < 0.001 for both comparisons). One-way ANOVA revealed a main effect of diet (*p* < 0.0001), starting at week 2, when KD was compared to both the NIH-31 and KS groups, depicted in Figure 2D.

After transitioning into new dietary groups, a significant decrease in blood glucose levels was found in KD-fed rats (one-way RM ANOVA, main effect of diet, *p* = 0.007, Figure 2E) together with increased peripheral levels of BHB (one-way RM ANOVA, main effect of diet, *p* < 0.0001, Figure 2F). Plasma insulin levels from rats fed KD were lower than other groups (*p* < 0.001 when compared to both NIH-31 and KS groups, week 1 time point, Figure 2G), showing a 50% reduction in comparison to the NIH-31-fed group (FLSD, *p* < 0.001). Ketone supplementation did not affect insulin levels when compared to the NIH-31 diet (FLSD, *p* = 0.21). On week 3, behavioral testing was performed to assess diet-induced changes in short-term memory and tail blood collection was not performed to avoid possible confounding variables in both behavioral and biochemical measures.

### 24 h retrieval in the novel object recognition test was enhanced in KD fed rats

**Figure 3:**
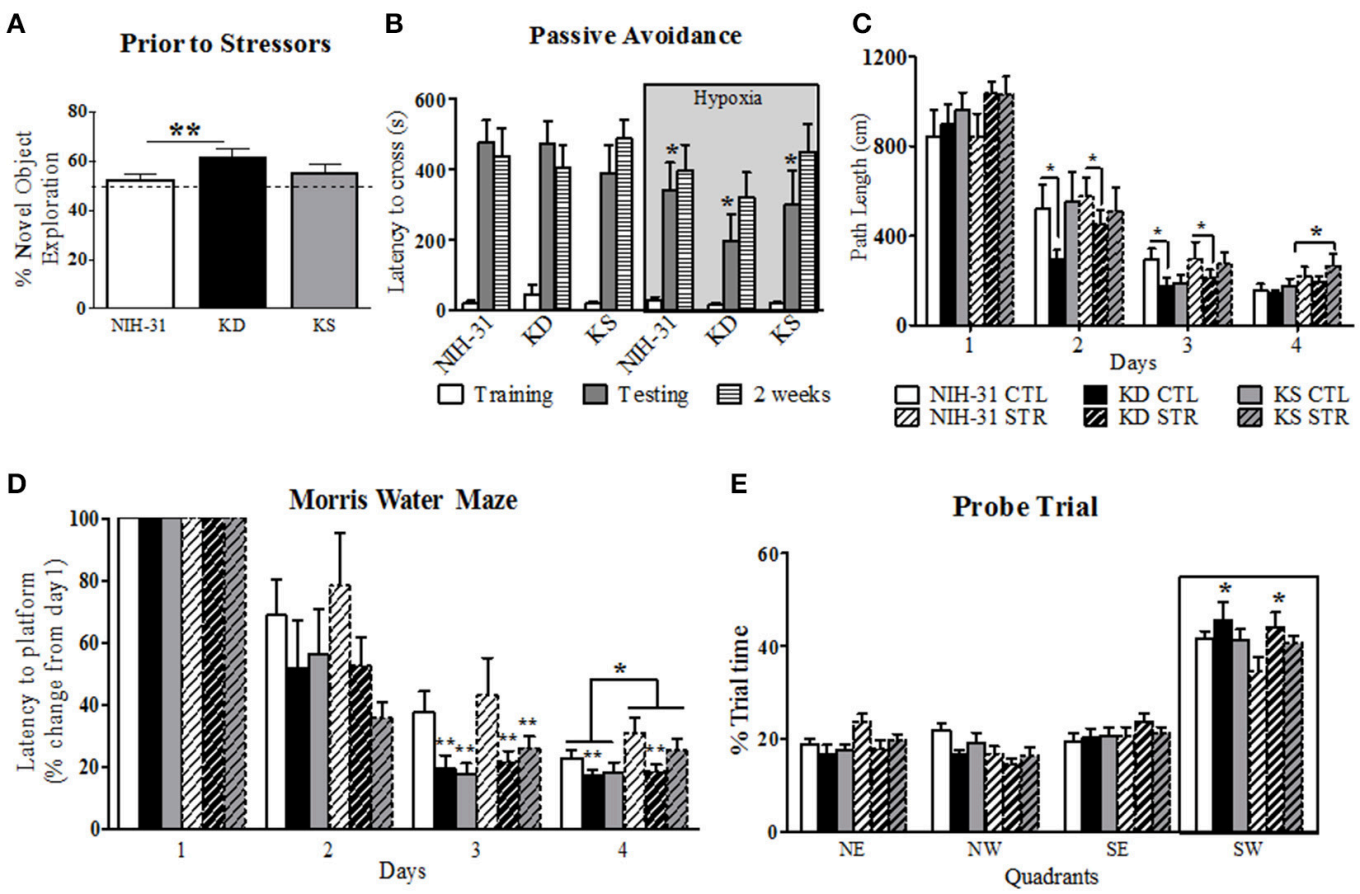
**Diet and stress-induced effects on behavioral performance prior to and during daily exposure to stressors. (A)** KD-fed rats showed greater percentage of novel object exploration compared to NIH-31 diet. This improvement occurred regardless of changes in locomotor activity (not shown). No differences in total distance traveled or speed were observed in any of the groups. The dotted line represents chance exploratory preference. **(B)** Hypoxia exposure prior to and following training trials significantly decreased latency to cross 24 h later (shadedbars) in the passive avoidance test. This impairment, however, was not present 2 weeks later (dashed bars). **(C)** KD-fed rats displayed shorter path lengths on days 2 and 3 of testing in the Morris water maze. Stress significantly increased path length on day 4 of testing, regardless of diet. **(D)** Morris water maze performance was significantly improved in both KD and KS groups on day 3, regardless of stress, and was impaired by stress on day 4. KD feeding prevented stress-induced impairments in performance on day 4 **(E)** Percentage of trial time spent in each quadrant during probe trial. KD-fed rats spent more time in target quadrant (SW) than NIH-31-fed groups. Data are presented as mean ± S.E.M. (*n* = 9–10/group). ^*^p ≤ 0.05; ^**^p < 0.01.

Short-term memory was assessed by the novel object recognition test. One-way ANOVA revealed a main effect of diet (*p* = 0.03); Figure 3A highlights the *post-hoc* comparison showing that the KD-fed group exhibited significantly greater percentage of novel object exploration compared to NIH-31-fed group (FLSD, *p* = 0.009). No differences were observed between KD-fed and either KS (FLSD, *p* = 0.11) or between KS vs. NIH-31 (FLSD, *p* = 0.29) groups.

### 24 h performance in the passive avoidance was negatively affected by hypoxia

The passive avoidance test was used to investigate learning and memory performance following exposure to stress induced by exposure to hypoxia prior to and immediately after training trials. All animals entered the dark chamber rapidly on training day and two-way ANOVA revealed no diet (*p* = 0.74) or hypoxia (*p* = 0.63) differences when latency to cross was analyzed (Figure 3B, clear bars). Three-way RM ANOVA (diet, hypoxia and testing session) revealed main effects of hypoxia (gray box, *p* = 0.05), testing session (*p* < 0.0001) and significant interaction between testing session and hypoxia (*p* = 0.02). Specifically, groups that were exposed to hypoxia showed significantly shorter latencies to cross over to the dark chamber when compared to animals kept in normoxic conditions during testing session (two-way RM ANOVA *p* = 0.01), indicating poorer performance. This detrimental effect was, however, no longer present when animals were tested 2 weeks later (*p* = 0.45; Figure 3B, striped bars). There were no significant effects of diet during training, 24 h or 2 weeks testing for the passive avoidance test (three-way RM ANOVA, *p* = 0.61).

### Performance in the MWM was modulated by both diet and stress

Spatial learning and memory were assessed by the MWM after multi-modal stress exposure. All animals swam without difficulty and no differences in swim speed were observed between diets or stress (*p* = 0.95 and *p* = 0.92, respectively, data not shown). A non-significant trend was observed for a main effect of stress on path length (*p* = 0.07), suggesting that exposure to stress resulted in a trend for increased swim distance. This effect reached statistical significance on day 4 (two-way ANOVA, main effect of stress, *p* = 0.02, Figure 3C). Individual analysis of daily path length revealed that KD-fed rats swam significantly shorter distances when compared to NIH-31-fed rats on both days 2 and 3 (*p* = 0.05 and *p* = 0.04, respectively) regardless of exposure to stressors.

As evidenced by Figure 3D, all groups improved with gradual decreases in latency to reach the escape platform (three-way RM ANOVA main effect of days, *p* < 0.0001). Three-way ANOVA revealed a main effect of stress (*p* = 0.05), although no main effects of diet (*p* = 0.22) were observed when averaged latencies were compared across all 4 days. Stress-induced increase in latency to reach platform was most pronounced on day 4 of testing (*p* = 0.02). Significant differences between groups were observed within days when latency to reach platform on testing days was compared to initial performance on Day 1 (three-way RM ANOVA main effect of diet, *p* = 0.003). For instance, on Day 2 groups fed control diet showed a non-significant trend for increased latencies when compared to both KD (FLSD, *p* = 0.1) and KS groups (FLSD, *p* = 0.06). This difference reached statistical significance on Day 3 (main effect of diet, *p* = 0.005), with KD and KS groups displaying shorter latencies than control diet groups (FLSD, *p* = 0.004, and *p* = 0.006, respectively). Main effects of both diet (*p* = 0.03) and stress (*p* = 0.05) were observed on Day 4. KD-fed animals displayed significantly shorter latencies compared to the control diet (FLSD, *p* = 0.008, Figure 3D).

All animals spent the majority of probe testing time in the target quadrant (outlined by boxed area in Figure 3E). No differences in swim speed or distance were observed (data not shown). Three-way RM ANOVA (diet, stress, and quadrants) revealed a main effect of quadrant (*p* < 0.0001) and a significant interaction between quadrant and diet (*p* = 0.03) vs. a non-significant trend for quadrant and stress interaction (*p* = 0.07). KD-fed animals spent significantly more time in the target quadrant than NIH-31-fed groups (FLSD, *p* = 0.02).

### Neither diet affected hypothalamic-pituitary-adrenal axis response to acute stressor

**Figure 4:**
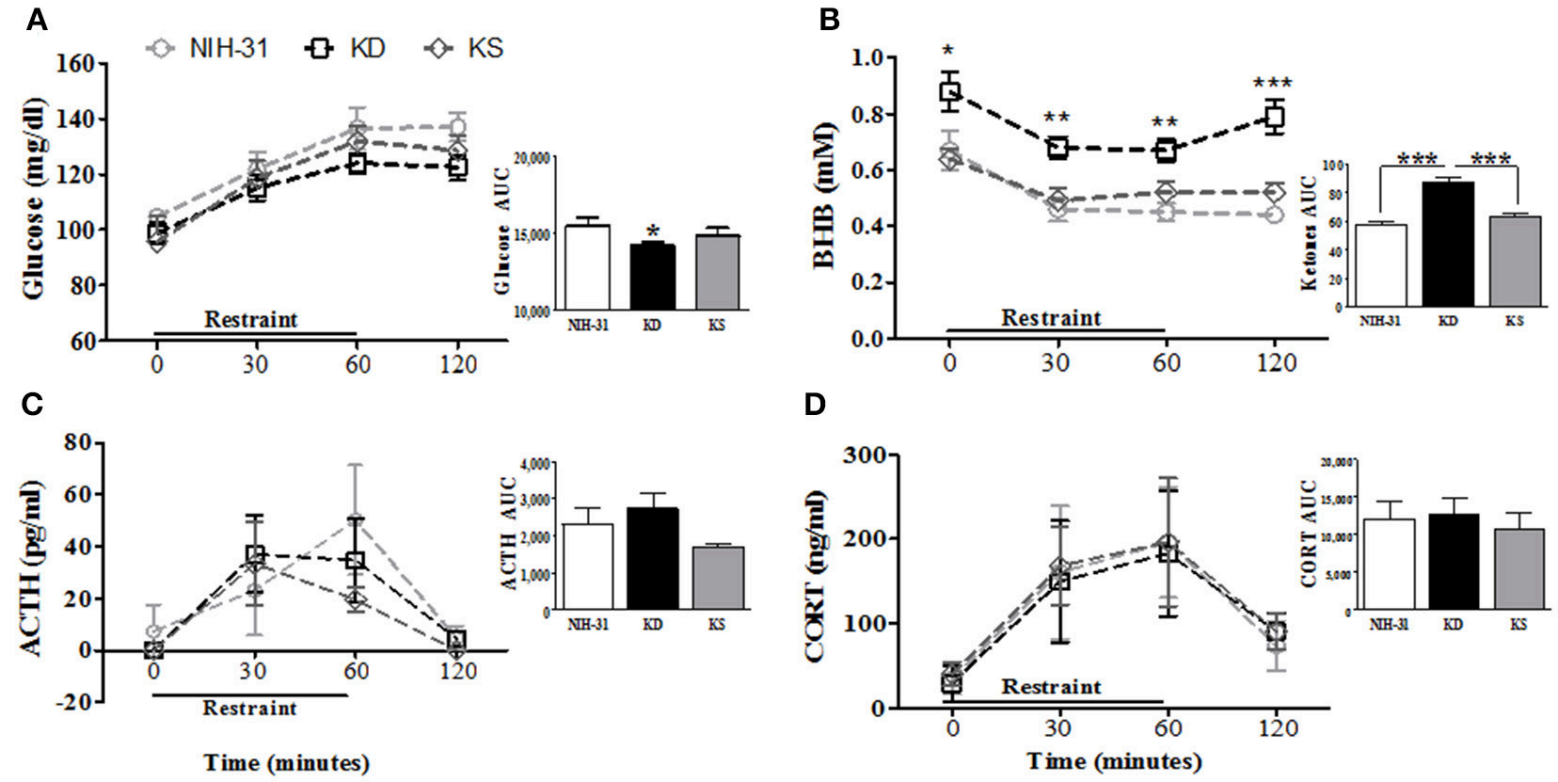
**Effects of endogenous (KD) vs. exogenous (KS) ketones on blood chemistry and the stress response. (A)** Blood glucose and **(B)** ketone levels during and following 1 h restraint challenge (*n* = 9–10/group). Plasma levels of the stress hormones **(C)** ACTH and **(D)** corticosterone were unchanged by either KD or KS. Insets represent area under the curve (AUC) analysis for each measurement. Data shown as mean ± S.E.M. (*n* = 6–8/group). ^*^p < 0.05, ^**^p < 0.01 and ^***^p < 0.001.

Diet-induced changes in stress hormone levels were assessed by a 1 h restraint challenge, during which blood samples were collected at 0 (baseline), 30 and 60 min and then after 1 h recovery (120 min). Figure 4 summarizes restraint-induced changes in peripheral levels of glucose (Figure 4A), BHB (Figure 4B), ACTH (Figure 4C), and CORT (Figure 4D). Glucose levels steadily increased (two-way RM ANOVA main effect of time *p* < 0.0001, Figure 4A) but no main effects of diet were observed (*p* = 0.15). Area under the curve analysis (AUC, Figure 4A inset) showed that KD group exhibited lower overall levels of glucose than NIH-31 group (*p* = 0.05). Blood glucose levels in KS group were intermediate and did not differ from either NIH-31 or KD groups (AUC analysis: FLSD, *p* = 0.28 and *p* = 0.36, respectively). Two-way RM ANOVA revealed main effects of time (*p* < 0.0001) and diet (*p* < 0.0001) when ketone levels were analyzed throughout all time points, suggesting that ketone levels gradually dropped after the onset of the stressor, concomitantly with the increase in glucose levels. *Post-hoc* analysis showed that this difference was due to KD-fed group presenting higher levels of ketone bodies at all time points tested when compared to other dietary groups [Figure 4B: KD vs. NIH-31, FLSD baseline (*p* = 0.02), 30 min (*p* = 0.001), 60 min (*p* = 0.0002) and 120 min (*p* < 0.0001); KD vs. KS, FLSD baseline (*p* = 0.01), 30 min (*p* = 0.004), 60 min (*p* = 0.007) and 120 min (*p* = 0.0001)]. Accordingly, area under the curve analysis (Figure 4B inset) revealed a main effect of diet (*p* < 0.001 and FLSD, *p* < 0.001) when compared to both groups.

No differences were observed in the total levels or area under the curve analysis for ACTH (Figure 4C and inset) or CORT (Figure 4D and inset) between groups. ACTH secretion profile in both KD and KS groups seemed slightly different, with peak levels appearing somewhat faster (at 30 min) and lower than control counterparts. However, these differences did not reach statistical significance which may be due in part to great individual variability in the biochemical measurements of ACTH in plasma samples.

### Postprandial levels of glucose, ketones, and insulin

**Figure 5:**
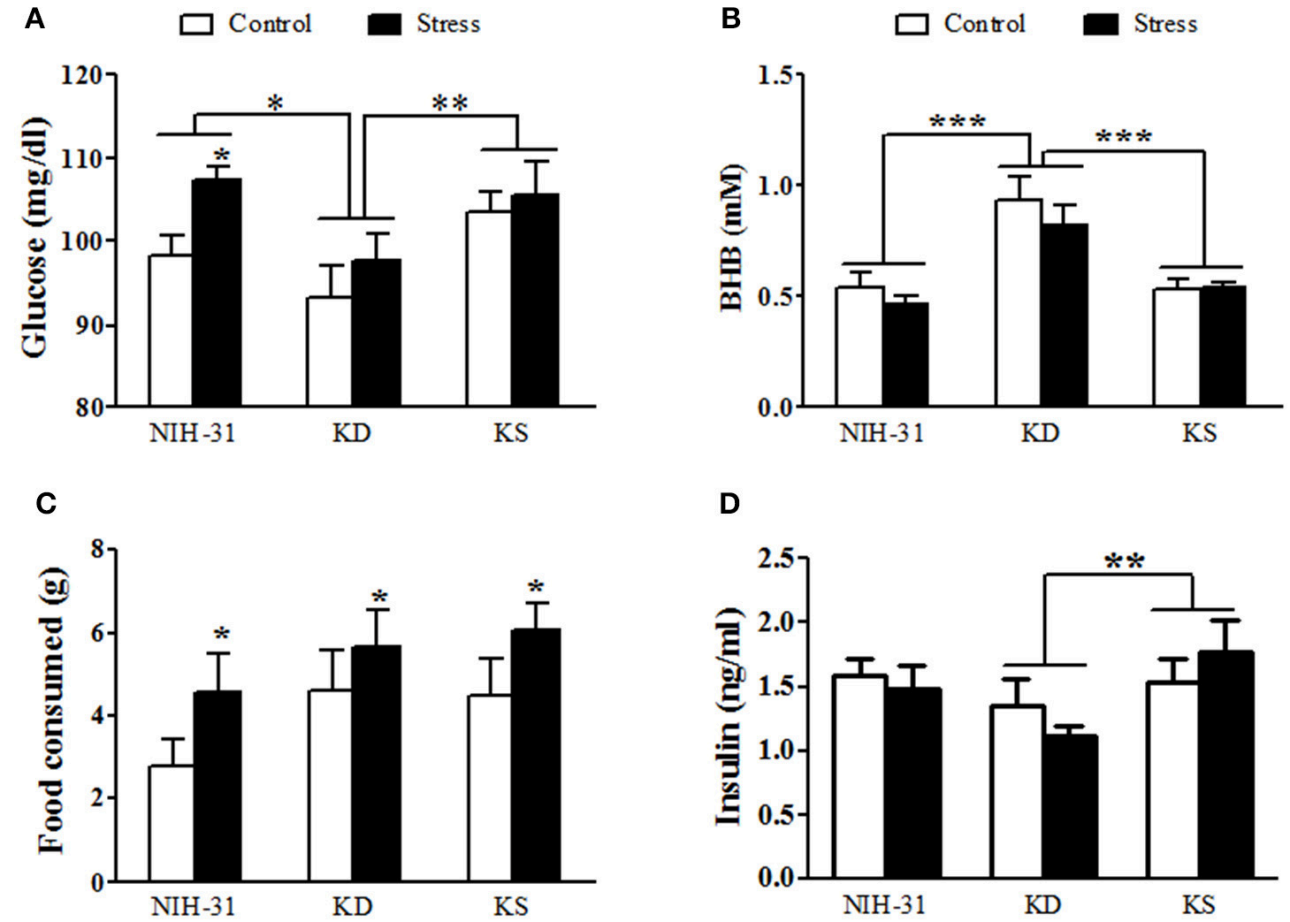
**Postprandial measurements of glucose, ketones, food intake and insulin. (A)** Blood glucose was reduced in KD-fed groups and elevated in stressed groups. **(B)** Ketone (BHB) levels were elevated in KD groups only and not affected by stress. **(C)** Food intake was not different across diets but was elevated in stressed groups. **(D)** Non-fasting insulin levels were reduced by KD. Data shown as mean ± S.E.M. (*n* = 8–10/group). ^*^p < 0.05, ^**^p < 0.01, and ^***^p < 0.001.

In order to determine diet-induced metabolic changes, all animals had food removed for 4 h (0800–1,200) followed by *ad libitum* access to respective diets for 2 h (1,200–1,400), after which food consumption was calculated and blood was collected for assessments of glucose, ketones and plasma insulin levels (Figure 5). Two-way ANOVA revealed main effects of both diet (*p* = 0.01) and stress (*p* = 0.04) on glucose levels 2 h after feeding. Stressed rats had greater postprandial glucose levels than control rats (FLSD, *p* = 0.03). Consistent with previous measurements, postprandial glucose measurements were attenuated in KD groups in comparison to both NIH-31 (FLSD, *p* = 0.01) and KS (FLSD, *p* = 0.004) groups (Figure 5A). Postprandial levels of BHB were increased in KD-fed group only (two-way ANOVA main effect of diet, *p* < 0.0001; FLSD, *p* < 0.0001 when compared to both other diets; Figure 5B), regardless of stress (*p* = 0.26). Of note, at the time point investigated, consumption of ketone supplements in the context of a normal carbohydrate diet was not effective in inducing significant ketosis when compared to NIH-31 diet alone (FLSD, *p* = 0.53). Food consumption was not affected by diet (*p* = 0.14) but was significantly increased in stressed groups (*p* = 0.04, Figure 5C).

Non-fasting insulin levels revealed a main effect of diet (*p* = 0.04, Figure 5D). *Post-hoc* comparisons showed that this effect was due to a pronounced decrease in insulin levels in KD-fed groups when compared to KS-fed (FLSD, *p* = 0.01) while displaying a non-significant trend in comparison to NIH-31-fed groups (FLSD, *p* = 0.10).

### Neither dietary treatment prevented body weight changes during chronic stress

**Figure 6:**
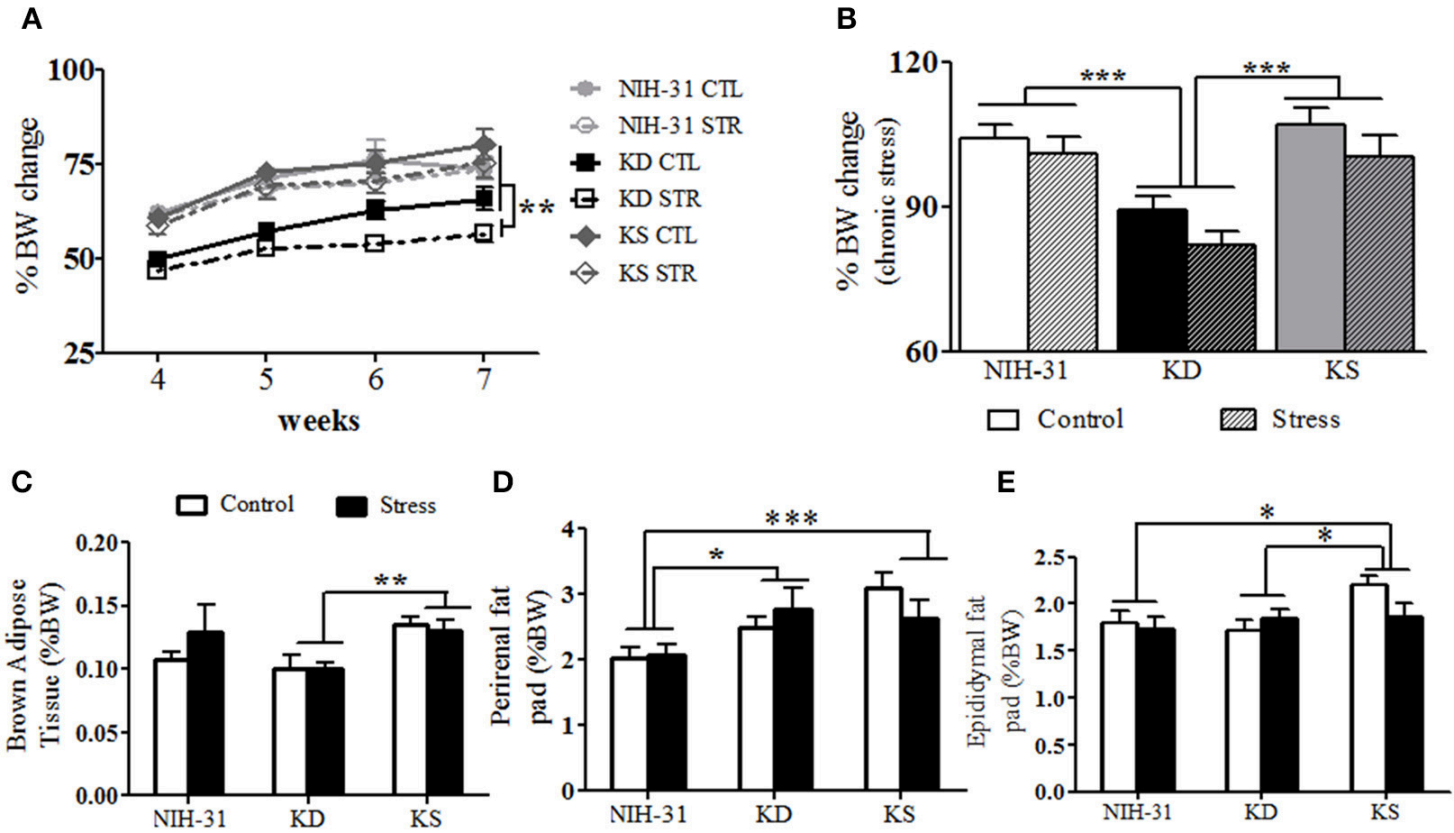
**Anatomical changes at euthanasia following chronic exposure to stressors. (A)** Following exposure to stressors, main effects of diet (*p* < 0.0001) and stress (*p* = 0.04) were observed, with KD-fed rats exposed to stress displaying significantly lower body weight changes. **(B)** Averaged percentage of body weight change values during the stress exposure period showed main effects of diet (*p* < 0.001) and stress (*p* = 0.04) and showed that KD groups showed consistent lower weight gain. **(C)** KS-fed rats showed increased brown adipose tissue weight (BAT, normalized to body weight) in comparison to KD-fed group (*p* = 0.002). **(D)** Normalized perirenal fat pad weights were larger in both KD and KS in comparison to NIH-31-fed groups (*p* = 0.01 and *p* = 0.0006, respectively). **(E)** A trend for main effect of diet (*p* = 0.07) was observed with normalized epididymal fat pad weights, with increased weights in KS groups, compared to both NIH-31 (*p* = 0.03) and KD (*p* = 0.04) groups. Data represented as mean ± S.E.M. (*n* = 9–10/group). ^*^p < 0.05; ^**^p < 0.01, and ^***^p < 0.001.

Weekly assessments (three-way RM ANOVA with diet, stress and weeks as independent variables) showed main effects of both stress (*p* = 0.04) and diet (*p* < 0.0001). All groups exposed to stressful conditions displayed smaller body weight changes throughout the 4 weeks of daily exposure to stressors (Figure 6A). The diet effect observed was due to KD-fed groups remaining significantly smaller than animals from the other dietary groups. Averaged body weight changes during the last 4 experimental weeks were analyzed to investigate the combined effects of both diet and stress (Figure 6B). Two-way ANOVA revealed main effects of both diet (*p* < 0.001) and stress (*p* = 0.04) on percentage of body weight change with *post-hoc* analysis indicating that KD-fed rats presented significantly smaller body weight changes when compared to both NIH-31 and KS diets (FLSD, *p* < 0.001 for both comparisons, Figure 6B). Importantly, chronic supplementation with ketones did not affect body weight gain in comparison to the control diet (FLSD, *p* = 0.75).

### Chronic effects of both diet and stress on anatomy

No differences in brain (diet: *p* = 0.12 and stress: *p* = 0.99), adrenal (diet: *p* = 0.39 and stress: *p* = 0.93) or spleen (diet: *p* = 0.90 and stress: *p* = 0.15) raw weights were observed. Thymus involution was observed in stressed animals, regardless of dietary group (*p* = 0.04). In addition to raw tissue weights, brown adipose tissue (BAT), perirenal, and epididymal fat pads are also shown as percentage of final body weight. Normalized BAT weight was significantly increased in KS groups (main effect of diet, *p* = 0.01, Figure 6C) and this effect was observed when compared to KD groups (FLSD, *p* = 0.0025). No stress-induced differences were observed in raw values (*p* = 0.87) or normalized BAT weight (*p* = 0.53). Perirenal and epididymal fat pads were dissected and weighed to assess chronic effects of diet and stress on peripheral energy deposits. Two-way ANOVA revealed a significant effect of diet on normalized perirenal fat pad weight (*p* = 0.002). Figure 6D highlights the *post-hoc* comparisons showing that perirenal fat pads obtained from NIH-31-fed animals were significantly smaller than the ones dissected from both KD (FLSD, *p* = 0.01) and KS (FLSD, *p* = 0.0006) groups. When analyzing normalized epididymal fat pad weights, a trend for diet-induced differences was observed (*p* = 0.07, Figure 6E). Overall, KS groups displayed larger epididymal fat pads when compared to both NIH-31 (FLSD, *p* = 0.03) and KD (FLSD, *p* = 0.05) groups. No stress effects were observed in either perirenal (*p* = 0.77) or epididymal (*p* = 0.38) fat pads, regardless of dietary treatment.

### Stress-induced reduction in hippocampal BHB and BDNF were attenuated by KD and both KD and KS, respectively

**Figure 7:**
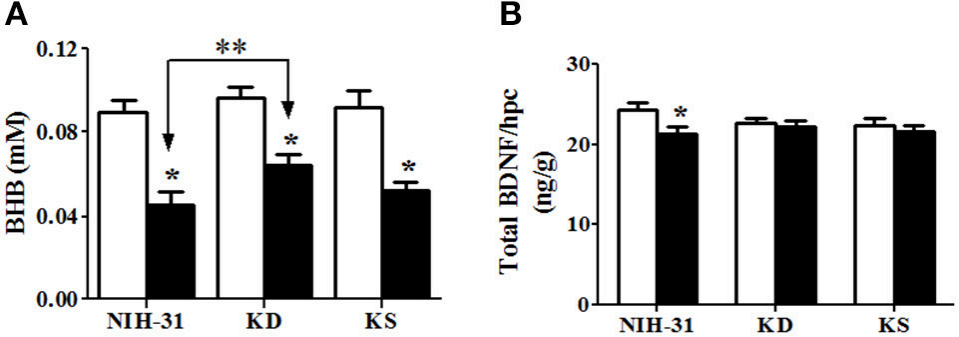
**Hippocampal levels of β-hydroxybutyrate (BHB) and brain derived neurotrophic factor (BDNF). (A)** Daily exposure to stress resulted in decreased hippocampal levels of BHB. This decrease, however, was attenuated in KD-fed rats compared with stressed rats on control diet (indicated by bracket with arrows). **(B)** Decreased BDNF levels were observed in stressed NIH-31 fed rats compared with non-stressed NIH-31 fed rats. However, both KD and KS groups showed a non-significant trend (*p* = 0.10) for lower levels of hippocampal BDNF. Data shown as mean ± S.E.M. (*n* = 8–10/group). ^*^p ≤ 0.05; ^**^p < 0.01.

Two-way ANOVA revealed a main effect of stress (*p* < 0.001) on hippocampal levels of BHB, with all diet groups showing lower levels following exposure to stressors (Figure 7A). While *post-hoc* comparisons did not find differences between any of the dietary groups in the control condition, stressed animals fed KD showed significant attenuation of decrements in hippocampal BHB levels, when compared to stressed NIH-31-fed group (FLSD, *p* = 0.02). Figure 7B depicts changes in the ratio of BDNF levels to hippocampal weight. Two-way ANOVA revealed a main effect of stress (*p* = 0.03), while *post-hoc* comparisons showed that NIH-31 fed groups showed a 13% reduction BDNF levels after repeated exposure to stressors (FLSD, *p* = 0.008) but no effect of stress was observed in either the KD or KS groups (Figure 7B).

### Diet and stress-induced changes in the protein levels of mitochondrial enzymes and glucose transporters

**Figure 8:**
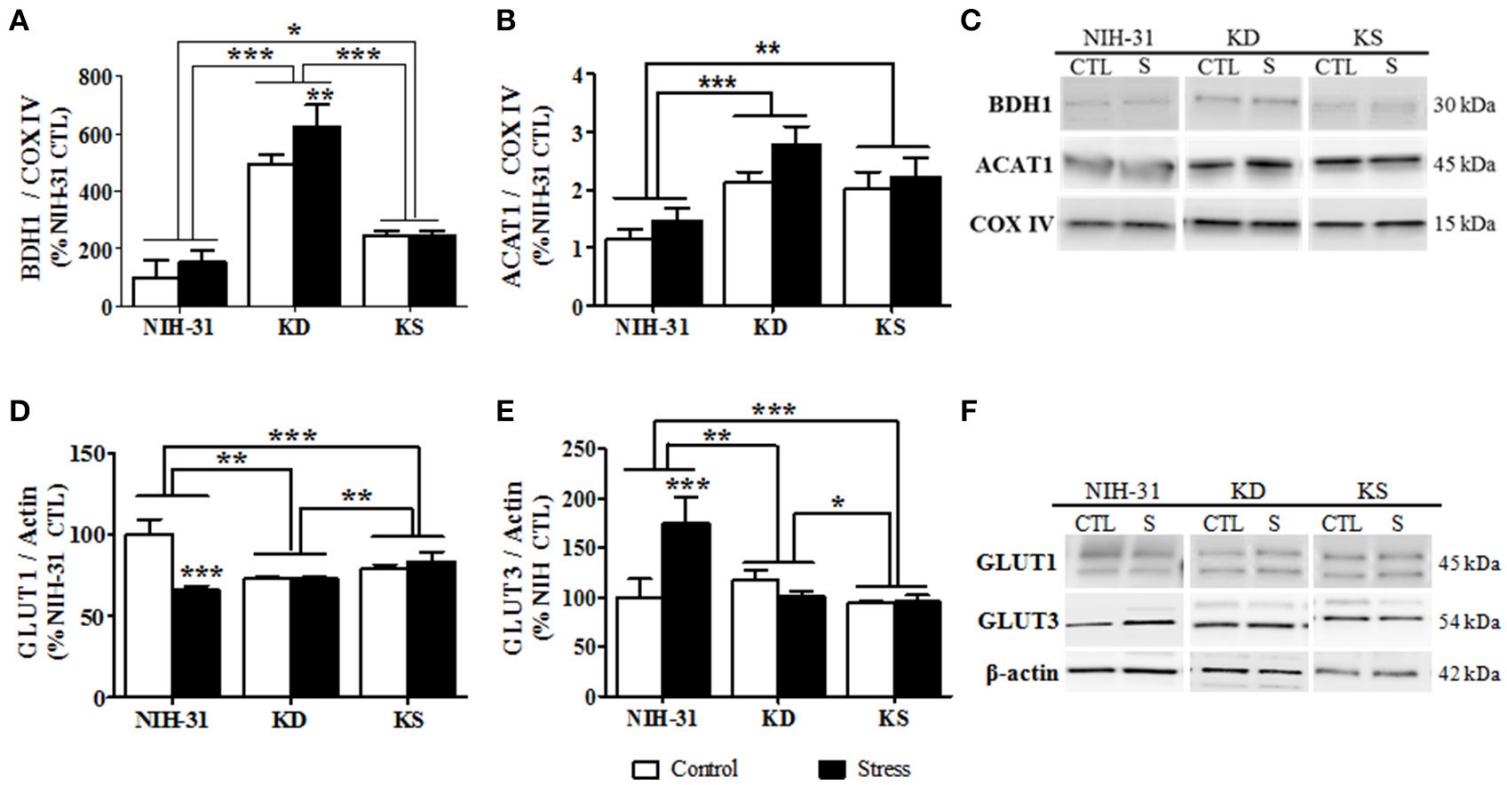
**Diet and stress-induced biochemical changes in mitochondrial enzymes and glucose transporters**. Hippocampal levels of the mitochondrial enzymes **(A)** 3-hydroxybutyrate dehydrogenase, type 1 (BDH1), and **(B)** acetyl-Coenzyme A acetyltransferase 1 following nutritional interventions and exposure to stressful conditions in healthy adult male rats. **(C)** Representative blots and corresponding loading control (COX IV). Diet and stress-induced changes in the levels of glucose receptors GLUT1 **(D)** and GLUT3 **(E). (F)** Representative immunoblots used and loading control (β-actin). Data shown as mean ± S.E.M. (*n* = 8–10/group). ^*^p < 0.05; ^**^p < 0.01, and ^***^p < 0.001.

β-hydroxybutyrate dehydrogenase-1 (BDH1), the enzyme that catabolizes BHB in the brain, was significantly increased in the hippocampi of rats fed either KD or KS (two-way ANOVA main effect of diet, *p* < 0.001, Figure 8A). After stress, a further increase was more prominent in KD when compared to both KS and NIH-31 groups (FLSD, *p* < 0.001 for both comparisons). *Post-hoc* comparison between KS and NIH-31 groups showed a significant increase in BDH1 levels (FLSD, *p* = 0.03). Despite the lack of main effects of stress, *post-hoc* analysis showed a significant increase in stress-induced levels of BDH1 levels in KD group (FLSD, *p* = 0.005). Figure 8B illustrates changes in acetyl-CoA transferase (ACAT1), a mitochondrial enzyme that catalyzes the reversible formation of acetoacetyl-CoA from two molecules of acetyl-CoA. Here we show that both endogenous and exogenous ketones induced a significant increase in hippocampal ACAT1 levels (main effect of diet, *p* < 0.001), when compared to control diet (FLSD, *p* < 0.001 and *p* = 0.003, respectively). Furthermore, a non-significant trend for a main effect of stress was observed (*p* = 0.06), mostly driven by the increase within KD-fed group (FLSD, *p* = 0.08). Figure 8C depicts representative blots for all groups probed with either BDH1 or ACAT1 and loading control (COX IV).

Two-way ANOVA revealed significant diet (*p* = 0.008), stress (*p* < 0.001) and diet and stress interaction (*p* < 0.001) in the levels of vascular endothelium glucose transporter (GLUT1, Figure 8D). Stress-induced decrease was only observed in the NIH-31 fed group (FLSD, *p* < 0.001). However, KD and KS groups displayed lower baseline levels of hippocampal GLUT1 when compared to NIH-31 group (FLSD, *p* < 0.001), regardless of stress exposure. Similarly, main effects of diet (*p* < 0.001), stress (*p* = 0.009) and diet × stress interaction (*p* < 0.001) were observed in the levels of neuronal glucose transporter (GLUT3, Figure 8E). Figure 8F depicts representative blots for all groups probed with either GLUT1 or GLUT3 and loading control (β-actin).

## Discussion

In this study, we investigated the effects of a ketogenic (KD) or ketone supplemented (KS) diet on metabolic, behavioral and biochemical outcomes associated with cognitive performance. Our results show that the KD, but not KS (at the dose tested here), elicited pronounced metabolic effects, and prevented some of the stress-induced effects on behavioral performance. Both KD and KS ameliorated decrements during day 3 of the water maze testing while improved performance during the probe trial was only observed in the KD group. During restraint stress, the KD group maintained peripheral ketosis despite increased glucose levels. The KD elicited biochemical changes in hippocampal levels of mitochondrial enzymes, glucose transporters and BDNF. Of interest, these biochemical changes were also observed in the KS group despite lack of peripheral ketosis and were not affected by exposure to stressors. Table 4 summarizes the main findings described in this report, showing that KD had effects on multiple outcomes examined in this study while the ketone supplemented diet studied was only able to replicate a portion of these observations.

To investigate the effects of nutritional treatments on behavior, all rats were tested on the novel object recognition tasks 3 weeks after transitioning into dietary groups and prior to starting the chronic stress paradigm. Our finding of increased novel object exploration in young subjects fed KD suggests that endogenous ketosis augmented short term memory acuity in healthy rats. Ketosis-induced improvement in the novel object recognition test had been previously reported in aged rats kept under normoxic conditions or after 1 day in hypobaric chambers (Xu et al., 2010). Subjects tested in our study did not show anxious or compulsive-like behaviors assessed by the marble burying test or time spent in the center of arena during novel object recognition testing (data not shown).

Next, we aimed to determine the impact of an acute restraint challenge on peripheral levels of stress hormones. Despite an overall increase in glucose, KD-fed animals sustained lower glucose levels (10% reduction in the area under the curve values) and greater ketosis throughout procedure, compared to both control and KS diets (52 and 38% higher, respectively). Even in the absence of statistically significance, subtle changes were observed in the profile of ACTH secretion, with a tendency for a shift in peak values and quicker return to baseline. These results were, nonetheless, not statistically significant and may benefit from further investigation using different dietary formulations, additional rodent species or continuous measurements to better investigate time course changes. Notably, several ACTH values from both KD and KS samples obtained during baseline and 120 min time points were below detection levels, whereas this was not observed with samples from rats fed the control diet. More studies are needed to better understand how changing cerebral energy metabolism to ketone utilization may affect the response to acute or chronic stressors.

Animals completed two behavioral tasks to determine whether the nutritional interventions adopted would prevent or diminish stress-induced detriments. Altitude sickness due to hypoxic conditions resulted in impaired performance on cognitive tasks in humans (Virues-Ortega et al., 2006) while KD showed neuroprotective properties following 3 weeks of hypoxia (Puchowicz et al., 2005, 2008). Neither KD nor KS prevented the hypoxia-induced impairment in the passive avoidance test. In fact, on testing day, animals on KD showed a slightly shorter latency to cross, suggesting that neuroprotective effects elicited by KD during hypoxic conditions may require prior adaptation, as evidenced by changes in cerebral energy utilization described in Puchowicz et al. (2005).

Previous investigations of KD-induced performance in the water maze have shown protective (Kim et al., 2012), detrimental (Zhao et al., 2004), or inconclusive (Fukushima et al., 2015) effects. Our findings reveal beneficial effects of KD despite repeated exposure to stressors known to elicit detriments in behavioral performance. Surprisingly, these effects were also observed in KS groups on the third day of testing; suggesting that supplementation with ketones may modulate biological pathways relevant to cognitive outcomes, despite lack of observable peripheral effects. Although, stress-induced increases in escape latency were observed, these effects only reached statistical significance on the fourth testing day and were prevented by KD feeding, suggesting that endogenous ketones positively impacted adaptation to the cumulative effect of stress on performance in the water maze. Several mechanisms have been suggested to explain ketone bodies effects on cognition. For instance, brain uptake of ketone bodies is proportional to their circulating levels (Blomqvist et al., 2002; Cahill, 2006), providing a more efficient fuel (Veech et al., 2001) for neuronal and glial cells and bypassing possible deficits in glucose uptake and utilization, prevalent in cases of neurodegenerative diseases (Mosconi et al., 2008), or during challenging situations (McNay et al., 2000).

Ketone bodies have also been reported to increase mitochondrial efficiency and biogenesis (Bough et al., 2006; Kashiwaya et al., 2010), which may also contribute to improved cerebral energy metabolism. Furthermore, ketones have also shown to act as potent signaling molecules, modulating energy metabolism (Marosi et al., 2016), epigenetic events (Shimazu et al., 2013) and neuronal excitability (Masino et al., 2011) (for a review, see Newman and Verdin, 2014b). Accordingly, the novel finding that KD attenuated stress induced decrease in hippocampal BHB levels may suggest that increased cerebral availability of ketone bodies plays a role in brain homeostatic mechanisms, modulating hippocampal energy utilization.

The KD diet was chosen based on its high MCT content, knowing that MCT ingestion can rapidly increase liver production of ketone bodies. Moreover, the MCTs included were chosen with the intent to induce neuroprotective effects, replacing standard saturated and hydrogenated fats present in other commercially available rodent KDs. The combination of canola and flaxseed oil was chosen to generate a 2:1 omega-3 to omega-6 ratio, a ratio suggested to be beneficial in well-formulated ketogenic diets. Given the neuroprotective properties associated with flaxseed oil (rich in 18:3 α-linolenic acid) (reviewed in Piermartiri et al., 2015), we acknowledge that the possibility remains that the increased levels of α-linolenic acid generated could also have contributed to the cognitive improvements observed, independently of the ketone bodies.

Hippocampal BDNF levels and expression can be upregulated following interventions that activate hormetic pathways (voluntary exercise, calorie restriction and environmental enrichment) (Mattson, 2008) or down regulated following chronic stress (for a review, see Rothman and Mattson, 2013). Our findings confirmed stress-induced decrements in hippocampal BDNF levels; however, this effect was only significant in NIH-31 fed rats. Due to its effect on decreased hippocampal excitability (Bough et al., 2003; Kawamura et al., 2014), KD is suggested to reduced BDNF signaling on the brain (Masino and Rho, 2012). This is in contrast with compelling evidence of up regulation of BDNF by strategies that, similarly to KD, reduce glycolytic activity such as caloric restriction (Stranahan et al., 2009) and 2-deoxy glucose treatment (Yao et al., 2011). Although, not statistically different, our findings are indicative of lower BDNF levels in both KD and KS control groups (7 and 8% lower, respectively). One other study described that feeding Wistar rats a KD for 8 weeks reduced BDNF levels in the striatum, but not the hippocampus (Vizuete et al., 2013).

Chronic administration of exogenous ketones via diet did not result in physiological levels of ketone bodies despite pronounced effects following acute administration. Intragastric administration of ketone supplements for 28 days resulted in rapid and sustained elevation of ketone bodies and decreased glucose levels in the absence of changes in lipid biomarkers (Kesl et al., 2016). Given that daily oral administration by gavage is a stressful method, we sought translatable approaches for human applications. Higher doses of ketone supplements may have successfully modulated peripheral metabolism considering the route of administration adopted in this study. However, incorporating large amounts of ketone supplements into one's diet may not be a feasible alternative, resulting in adverse gastrointestinal effects in addition to being a less palatable and more costly approach.

One advantage of choosing *ad libitum* feeding is that animals in all groups were able to decide how much food they wanted to eat and were not given an additional stressor (limited food availability), which would have been a confound to interpreting our results with chronic stress. We do, however, acknowledge that pair-feeding comparisons between KD and KS groups might address some of the differences reported in our study. Of note, considerable evidence from the literature has reported that KD-fed animals gain less weight despite showing no differences in food intake (Brownlow et al., 2013; Poff et al., 2013; Srivastava et al., 2013). We suggest that the changes described in our study are due to the dietary composition of each diet though the possibility remains that differences in energy intake contributed to our observed results. Although, food intake was not measured, body weight changes were similar between NIH-31 and KD-fed groups suggesting similar total caloric intake between these groups. Moreover, we observed a lack of diet-induced differences in food consumption following 4 h fasting. Exposure to stress, however, increased food intake following a brief period of food withdrawal (Figure 5C).

Overall, repeated exposure to stressors resulted in classical biochemical, behavioral and anatomical changes. Accordingly, hallmark features of the general adaptation syndrome (Selye, 1976) were seen with: increased energy expenditure suggested by reduced body weight gain despite increased food intake and thymus involution (Table 3). Neither diet was effective in preventing these outcomes, suggesting a possible disconnect between physiological mechanisms underlying the beneficial behavioral outcomes observed. Indeed, ketone supplementation resulted in behavioral improvements (shorter escape latencies on day 3 of MWM testing) and anatomical alterations (changes in peripheral energy deposits such as BAT, perirenal, and epididymal fat pads) being observed even in the absence of peripheral ketosis.

**Table 3:** **Brain and peripheral tissue weights at euthanasia**.

	**NIH-31**		**KD**		**KS**	
**Tissue**	**Control (10)**	**Stress (9)**	**Control (10)**	**Stress (10)**	**Control (10)**	**Stress (9)**
Brain	2.24 ± 0.03	2.23 ± 0.01	2.20 ± 0.02	2.22 ± 0.02	2.20 ± 0.02	2.19 ± 0.01
Adrenal	0.06 ± 0.002	0.06 ± 0.003	0.06 ± 0.04	0.07 ± 0.006	0.06 ± 0.002	0.06 ± 0.005
Thymus	0.37 ± 0.03	0.32 ± 0.05^*^	0.36 ± 0.03	0.32 ± 0.02^*^	0.43 ± 0.03	0.35 ± 0.03^*^
Spleen	0.89 ± 0.04	0.81 ± 0.04	0.86 ± 0.04	0.83 ± 0.03	0.87 ± 0.05	0.85 ± 0.03
BAT	0.53 ± 0.03	0.61 ± 0.09	0.48 ± 0.04^*^^,^ ^###^	0.47 ± 0.02^*^^,^ ^###^	0.69 ± 0.04^*^	0.64 ± 0.04^*^
Perirenal	10.04 ± 0.89	10.03 ± 0.82	12.11 ± 0.97	12.97 ± 1.54	16.15 ± 1.36^***^	13.39 ± 1.88^***^
Epididymal	8.88 ± 0.63^#^	8.34 ± 0.61^#^	8.40 ± 0.74^#^	8.73 ± 0.51^#^	11.35 ± 0.74	9.32 ± 1.01
BAT (%BW)	0.11 ± 0.005	0.13 ± 0.02	0.10 ± 0.01^##^	0.10 ± 0.005^##^	0.13 ± 0.006	0.13 ± 0.09
Perirenal (%BW)	2.03 ± 0.17	2.08 ± 0.18	2.49 ± 0.17^§^	2.77 ± 0.33^*^	3.12 ± 0.21^***^	2.63 ± 0.28^***^
Epididymal (%BW)	1.80 ± 0.12^Δ^	1.73 ± 0.12^Δ^	1.72 ± 0.12^Δ^	1.85 ± 0.11^Δ^	2.20 ± 0.10	1.86 ± 0.16

Thymus: ^*^Stress effect (p < 0.05).

Brown Adipose Tissue (BAT): Diet effect (p < 0.001), ^*^different than NIH-31 (p < 0.05);

### *different than KS (p < 0.001)*. Perirenal fat pad: Diet effect (p < 0.01), ^***^different than NIH-31 (p < 0.001).

*Epididymal fat pad: Diet effect (p < 0.05)*,

# *different than KS (p < 0.05)*.

*BAT, (%BW): Diet effect (p < 0.05)*,

## *different than KS (p < 0.01)*.

*Perirenal (%BW): Diet effect (p < 0.01), different than NIH-31*,

* *(p < 0.05)*,

*** *(p < 0.001).*;

§ *different than both NIH-31 and KS control groups (p < 0.05)*.

*Epididymal (%BW): Trend for diet effect (p = 0.07)*,

Δ *different than KS (p < 0.05)*.

**Table 4:** **Summary of main findings**.

				**DIETS**					
		**Parameters**		**NIH-31**		**KD**		**KS**	
Baseline (wk 0–3)	Metabolism	BW (wks 0–3)		–		↓↓↓^*^		–	
		Glucose (wk 1)		–		↓↓^*^		–	
		BHB (wk 1)		–		↑↑↑^*^		–	
		Insulin (wk 1)		–		↓↓↓^*^		–	
		Food intake		–		–		–	
		ACTH		–		–		–	
		CORT		–		–		–	
	Behavior	% Exploration novel object (24 h) (wk 3)		–		↑^*^		–	
				**Control**	**Stress**	**Control**	**Stress**	**Control**	**Stress**
Stress (wk 4–8)	Behavior	Passive Avoidance (wk 4)	24 h	–	↓^ˆ^	–	↓^ˆ^	–	↓^ˆ^
			2 wks	–	–	–	–	–	–
		MWM (wks 5,6)	Path length	–	↑^ˆ^	–	↓^*^	–	↑^ˆ^
			Latency	↓	↑^ˆ^	↓	↓↓^*^	↓	↑^ˆ^
			Probe	↑	↓^ˆ^	↑	↑^*^	↑	↓^ˆ^
	Biochemistry	BHB		–	↓↓↓^ˆ^	–	↓^*^	–	↓↓↓^ˆ^
		BDNF		–	↓^ˆ^	–	–	–	–
		BDH1		–	–	↑↑↑^*^	↑↑↑^*^^ˆ^	↑↑↑^*^	↑↑↑^*^
		ACAT1		–	–	↑↑↑^*^	↑↑↑^*^^ˆ^	↑^*^	↑^*^
		GLUT1		–	↓↓↓^ˆ^	↓↓↓^*^	↓↓↓^*^	↓↓↓^*^	↓↓↓^*^
		GLUT3		–	↑↑↑^ˆ^	↓↓^*^	↓↓^*^	↓^*^	↓^*^
	Organ weight at Euthanasia	Body weight		–	↓^ˆ^	↓^*^	↓↓^*^^ˆ^	–	↓^ˆ^
		Brain weight		–	–	–	–	–	–
		Adrenals		–	–	–	–	–	–
		Thymus		–	↓^ˆ^	–	↓^ˆ^	–	↓^ˆ^
		Spleen		–	–	–	–	–	–
		BAT		–	–	–	–	↑^*^	↑^*^
		Epididymal fat pad		–	–	–	–	↑^*^	↑^*^
		Perirenal fat pad		–	–	↑↑^*^	↑↑^*^	↑↑↑^*^	↑↑↑^*^

↑ or ↓ (p < 0.05); ↑↑ or ↓↓ (p < 0.01) and ↑↑↑ or ↓↓↓ (p < 0.001)

* Main effect of Diet and

ˆ *Main effect of Stress*.

Ketone supplementation resulted in increased brown adipose tissue, although this difference only reached statistical significance in comparison to KD groups. This finding is in agreement with other groups claiming that exogenously delivered ketones resulted in increased resting energy expenditure and sympathetic activity (Srivastava et al., 2012; Veech, 2013), an effect not observed in KD groups. In rodents, increased adiposity has been reported in KD-fed rats (Kinzig and Taylor, 2009), an effect abolished in the presence of voluntary exercise (Kinzig et al., 2010). On the contrary, humans kept on KD display greater weight loss and reduced fat mass. This discrepancy highlights inherent challenges of utilizing rodent models. Lack of fidelity when translating rodent outcomes into human applications has been previously described (Martin et al., 2010; Hodge et al., 2016); thus, caution should be exercised and clinical studies remain necessary.

Next, we sought to investigate the interplay between stress and metabolic control of cerebral energy regulation by assessing hippocampal levels of mitochondrial enzymes involved in ketogenesis pathways and glucose transporters. Both KD and KS groups significantly upregulated key mitochondrial enzymes associated with the catalytic conversion and utilization of ketone bodies (BDH1 and ACAT1). This novel finding indicates that chronic administration of exogenous ketones may be capable of modulating brain energy pathways regardless of changes in peripheral metabolism. Furthermore, reduced GLUT1 levels following chronic stress observed in control diet group was not present in either KD or KS groups. Taking into consideration that the capacity for glucose transport depends on the concentration of transporter proteins (Simpson et al., 2007), this decrease suggests an impairment in brain glucose availability.

Our findings of stress-induced increase in GLUT3 levels in control diet animals is in agreement with previous findings in rodents (Reagan et al., 1999), suggesting that stress may contribute to increased neuronal energy demands under conditions of high allostatic load. The increase in neuronal glucose transporter, coupled with a decrease in the levels of the vascular glucose transporter highlights a mismatch in energy availability/demand, likely underlying detriments in brain homeostasis following exposure to stressors. This effect was not observed in either dietary condition, suggesting that the presence of an alternative energy substrate may be efficiently buffering the stress-induced imbalance in hippocampal energetic demands. The observed changes in mitochondrial enzymes and glucose transporters resulting from both endogenous and exogenous ketones support the concept of ketone bodies being readily “pushed” into the brain in direct proportion to circulating levels vs. glucose being actively “pulled” depending on its utilization by neurons and astrocytes, described in Cunnane et al. (2016).

We chose to investigate whether a nutritional intervention increasingly used for its therapeutic properties augmented performance and mitigated challenge-induced deficits in healthy young rodents. Mixed results can often be attributed to methodological approach, such as: rodent model (different strains of rats or mice) (Ari et al., 2016), age (McNay and Gold, 2001), diet formulation and study length. For instance, metabolic effects of a western diet were more pronounced in Wistar rats when compared to Sprague-Dawley rats (Marques et al., 2016). Accordingly, *in vitro* hippocampal slices from Sprague-Dawley rats treated with a mixture of ketone bodies failed to show antiseizure effects or changes in synaptic transmission when using extracellular glucose concentrations commonly used by acute physiological recordings (Thio et al., 2000; Youssef, 2015), whereas other studies have found significant differences using different rodent models or by changing extracellular glucose concentrations during incubation and recording of slices (for a review, see Kawamura et al., 2016).

Furthermore, different ketone supplements, supplement mixture, dosage or administration route could have also impacted our findings and further studies need to be carried out for optimization purposes. Despite these potential differences in design, our study was able to detect changes in metabolic, behavioral and biochemical parameters associated with cognitive performance in both control and stress conditions in the ketone supplemented group.

Our results highlight the dissociation between metabolic, behavioral and biochemical outcomes reported, with the novel finding that exogenous ketones mimic KD changes in both mitochondrial enzymes and glucose transporters. The exogenous ketone mixture tested, when added to a normal carbohydrate content diet (at the dose adopted), were below detection limits in the periphery. However, changes to biochemical machinery associated with ketogenesis pathways and glucose uptake were modulated similarly by both dietary interventions. We hypothesize that, after digestion and absorption into circulation, the ketone bodies produced were available for brain uptake, possibly displacing glucose as the brain's preferred fuel (Veech et al., 2001; Lamanna et al., 2009) and leading to the biochemical effects described. This intriguing finding warrants further investigation as it is likely a promising mechanism by which brain adaptive responses can be modulated, leading to enhanced performance.

In conclusion, we describe here that endogenous ketosis affected metabolic and behavioral outcomes in both stressed and control conditions, whereas these results were only observed in part with the exogenous ketone supplementation protocol tested. However, we report that, in the hippocampus, both endogenous and exogenous ketones were effective in modulating biochemical parameters associated with metabolic and cognitive responses. Our study advances the current views on the subject of performance optimization through a nutritional approach using ketone bodies to modulate metabolic and cognitive outcomes. Taken together, our findings suggest that ketogenic diets and, to a lesser extent, ketone supplements can modulate brain adaptive responses mediating cognitive performance in healthy young subjects during both control or stressed conditions.

## Author contributions

Animal handling, behavioral assessments, and tissue collection were conducted at the WPAFB animal facility. Biochemical assays were performed in WPAFB research laboratories (AFRL). This study was conceptually conceived by MB and RJ. MB, RM, NB, and SJ contributed to the design of animal experimentation, such as scheduling of exposure to stressors and behavioral tasks. MB, RM, NB, and SJ participated in the acquisition, analysis, and interpretation of the data. All authors contributed to the drafting and critically revising the intellectual content included in this manuscript. Additionally, all authors have read and approved the final version of the manuscript.

## Funding

This work was supported by the Air Force Office of Scientific Research [14RH08COR]. MB is recipient of a postdoctoral fellowship from the National Research Council [FA9550-12-D-0001]. Neither sponsor had any participation in the study design collection, analysis or interpretation of data nor in the writing of this manuscript. Distribution statement: Approved for public release: distribution is unlimited. 88ABW Cleared 09/08/2016; 88ABW-2016-4390.

### Conflict of interest statement

The authors declare that the research was conducted in the absence of any commercial or financial relationships that could be construed as a potential conflict of interest. Personnel (RM, NB) from a government contracting company-Infoscitex a wholly owned subsidiary of DCS Corp. - provided support for this work, by participating in study design (establishing novel procedures, scheduling stressors, and behavioral testing), data collection, analysis, and critically revising the final submitted manuscript. Support from Infoscitex, Inc. pertains to contracting personnel to support research activities at the Air Force Research Laboratory and does not involve financial incentives, products, supplies, or biological applications that would influence our research questions or outcomes.
